# Exosome in Tumour Microenvironment: Overview of the Crosstalk between Normal and Cancer Cells

**DOI:** 10.1155/2014/179486

**Published:** 2014-05-21

**Authors:** Catarina Roma-Rodrigues, Alexandra R. Fernandes, Pedro Viana Baptista

**Affiliations:** ^1^Departamento de Ciências da Vida, Faculdade de Ciências e Tecnologia da Universidade Nova de Lisboa, Campus de Caparica, 2829-516 Caparica, Portugal; ^2^Centro de Química Estrutural, Complexo 1, Instituto Superior Técnico, Universidade de Lisboa, Avenida Rovisco Pais, 1049-001 Lisboa, Portugal; ^3^CIGMH, Departamento de Ciências da Vida, Faculdade de Ciências e Tecnologia da Universidade Nova de Lisboa, Campus de Caparica, 2829-516 Caparica, Portugal

## Abstract

Cancer development is a multistep process in which exosomes play important roles. Exosomes are small vesicles formed in vesicular bodies in the endosomal network. The major role of exosomes seems to be the transport of bioactive molecules between cells. Depending on the cell of origin, exosomes are implicated in the regulation of several cellular events, with phenotypic consequences in recipient cells. Cancer derived exosomes (CCEs) are important players in the formation of the tumour microenvironment by (i) enabling the escape of tumour cells to immunological system and help initiating the inflammatory response; (ii) acting in the differentiation of fibroblasts and mesenchymal cells into myofibroblasts; (iii) triggering the angiogenic process; and (iv) enhancing the metastatic evolution of the tumour by promoting epithelial to mesenchymal transformation of tumour cells and by preparing the tumour niche in the new anatomical location. Since the finding that exosomes content resembles that of the cell of origin, they may be regarded as suitable biomarkers for cancer diagnosis, allowing for diagnosis and prognosis via a minimal invasive procedure. Exosome involvement in cancer may open new avenues regarding therapeutics, such as vectors for targeted drug delivery.

## 1. Introduction

The major modifications to normal cells that result in the formation of a tumour derive from mutational events in oncogenes or tumour suppressor genes that occur in a normal cell and will ultimately lead to uncontrolled growth [1]. This is the case of cell cycle regulatory genes (e.g.,* RB1*), cell cycle checkpoint related genes (e.g.,* TP53*), genes encoding proteins involved in DNA integrity maintenance and in sustaining propagation of damaged cells (e.g.,* MLH1*,* MSH2*,* BRCA1* and 2), and genes involved in inhibition of apoptotic pathways (e.g., bcl2 overexpression) [2]. Evasion of growth and apoptotic control is usually followed by angiogenesis and metastasis. Many obstacles must be overcome, namely, the ability to survive an inhospitable microenvironment, where intercommunication between tumour cells and their surrounding microenvironment is essential for overcoming this obstacle and for tumour progression [3].

Despite the importance of the modifications occurring at the cell level, the tumour microenvironment is also relevant for the development of cancer. In fact, in the case of epithelial tumours, it is the combination of tumorigenic cells and stromal cells that dictates the extracellular matrix composition of the carcinoma [4]. The intracellular and intercellular communication between tumour and stromal cells is accomplished via cell-cell interactions (mediated by gap junction channels), paracrine mechanisms involving growth factors, chemokines, and proteases, as well as by extracellular vesicles [5]. Concerning the biological function of the vesicles involved in cell-cell communication, two main classes are considered: exosomes and microvesicles. These vesicles are secreted by most normal and malignant cells and share in common an enclosed lipid bilayer. However, while microvesicles are generated by budding from the plasma membrane, exosomes are derived from the endolysosomal pathway [5, 6]. Exosomes are involved not only in the cell-cell communication in “bulk” tumour microenvironment but also between tumour and distant cells, favouring secretion of growth factors, cytokines, and angiopoietic factors by stromal cells, induction of proliferation of endothelial cells, metastasis, and immune responses [7, 8]. Therefore, exosomes constitute valuable biomarkers for cancer diagnosis and prognosis and also constitute either targets or vectors for therapeutic approaches in cancer [9].

In this review we intend to highlight the relevance of exosomes in tumorogenesis, highlighting their biogenesis, composition, and main function, and then focusing on their role in cancer development and progression. Finally, we will address the potential of exosomes as biomarkers and their use for cancer therapy.

## 2. Exosomes Biogenesis

Exosomes are formed in the endosomal network. The formation of the early endosomes occurs in the plasma membrane by the fusion of endocytic vesicles [10]. The maturation process consists in an acidification of the endosome lumen, via altered protein content and fusion with intraluminal vesicles (ILVs), which are formed by invaginations of the endosomal membrane, randomly engulfing portions of the cytosolic contents. The process of ILVs formation requires specialised units highly enriched with tetraspanins (such as CD9, CD63, CD81, CD82, and CD151) and several complexes called endosomal sorting complex required for transport (ESCRT complex) [10]. The presence of phosphatidylinositol 3-phosphate, ubiquitinated cargos in early endosomes vesicles and the curved membrane topology of the vesicles, which is reached by protein-protein interactions of the tetraspanins, induces the recruitment of ESCRT-I and ESCRT-II [6]. These proteins, together with ESCRT-III, which binds ESCRT-I via the protein Alix, promote the budding of the membrane [6]. Furthermore, this process also involves protein-lipid interactions, including interaction of ESCRT proteins with oxysterols and polyglycerophospholipid BMP (bismonoacylglycerolphosphate) [11].

At the end of the maturation process, the multivesicular bodies (MVBs) composed of late endosomes together with ILVs, are situated close to the nucleus. The fusion of MVBs with the plasma membrane leads to the release of the ILVs to the extracellular environment, which are then referred to as exosomes. The releasing process of exosomes may be accomplished by the outward exosome and microvesicle budding pathway or by an inducible release, a highly regulated process that involves several components of the endocytic machinery, including the Rab GTPases, such as Rab11, Rab35, Rab27a, and Rab27b, cytoskeleton regulatory proteins, heparanase, and SNARES (soluble NSF attachment receptor) for target fusion [12–14]. An increased exosome release was found to be triggered by several types of stress, such as changes in pH membrane, hypoxia, oxidative stress, thermal changes, shear stress, and radiation, as well as by stimulation of sphingomyelinase and subsequent formation of ceramide and activation of the tumour suppressor protein p53 [7, 13, 15–17]. Additionally, a feedback regulatory mechanism for controlling exosome release in breast cancer cells was proposed, being observed that exosomes derived from cancer cells also inhibited the exosome release from normal breast cells and suggested a dominant regulatory effect of cancer cell derived exosomes (CCEs) [18].

## 3. Exosomes Composition

Exosomes are small vesicles ranging in size between 30 and 120 nm, composed by a lipid bilayer containing membrane proteins that surrounds a lumen comprising proteins and nucleic acids, that vary according to cell type and mechanism of biogenesis [19]. As an example, exosomes isolated from malignant effusions of cancer patients contain tumour specific proteins in their surface, such as Her2/Neu from ovarian cancer ascites and Mart1 from patients with melanoma [20]. Additionally, growth factors, such as tumour necrosis factor-alpha (TNF-*α*), epidermal growth factor (EGF), and fibroblast growth factor (FGF), have also been found associated with exosomes [21–23].

### 3.1. Protein Content of Exosomes

Over 4600 different proteins have been associated to exosomes, including proteins from the cytosol, the plasma membrane, Golgi apparatus, and endoplasmic reticulum [24, 25]. Due to a common biogenesis path, the most part of exosomes contain proteins involved in the endosomal network, including (i) membrane transport and fusion proteins, such as GTPases, annexins, Rab proteins, and flotillin; (ii) tetraspanins, such as CD9, CD63, CD81, and CD82; (iii) heat shock proteins (HSPs), such as Hsp60, Hsp70, and Hsp90; (iv) proteins involved in MVBs biogenesis, such as Alix and TSG101; (v) cytoskeletal proteins, such as actin, tubulin, syntenin, and moesin; and (vi) lipid-related proteins and phospholipases [7, 19, 26]. Additionally, metabolic enzymes, signal transduction proteins, the carrier protein albumin, and major histocompatibility complement antigens are also commonly found in exosomes [14]. Due to the higher frequency of these proteins, tetraspanins, Alix, flotillin, TSG101, and Rab5b have been frequently used as markers for identification and confirmation of the presence of exosomes [19].

Several studies have shown that CCEs can alter the extracellular matrix through secretion of matrix metalloproteinases (MMPs) or activators of MMPs, such as HSPs. MMPs are zinc-dependent plasma membrane endopeptidases that can degrade extracellular matrix proteins, such as collagen, proteoglycans, fibronectin, and laminins [7]. Hakulinen and collaborators [27] showed that fibrosarcoma and melanoma derived exosomes can secrete MT1-MMP able to activate pro-MMP2 and to degrade collagen and gelatine. Other studies have demonstrated that Hsp90 is also secreted via exosomes and can activate MMP2 to enhance invasion of cancer cells [28].

### 3.2. Lipid Content of Exosomes

The exosomal lumen is surrounded by a lipid bilayer enriched in (i) raft-associated lipids such as cholesterol; (ii) diglycerides; (iii) sphingolipids, such as sphingomyelin and ceramide; (iv) phospholipids; (v) glycerophospholipids, such as phosphatidylcholine (PC), phosphatidylserine (PS), phosphatidylethanolamine (PE), and phosphatidylinositol (PI); and (vi) polyglycerophospholipids [11]. Interestingly, the content of lipids in exosomes differs substantially from that of the parental cells. While the content of sphingomyelin, PS, PI, ceramides, and cholesterol is highly increased in exosomes, the content of PC is decreased (except in reticulocytes) [7, 11]. Additionally, despite similarities of the transmembrane orientation between exosomes and parental cells, the exosomal PS is found to be randomly distributed between the two membrane leaflets, with an enrichment in the external exosomal membrane, contrary to the viable parental cell membrane, where it is located in the inner leaflet [11]. In general, this lipid composition confers rigidity to the vesicle, which confers stability of exosomes in biological fluids and cell culture mediums [11]. Presence of PS on the outer membrane of exosomes can function in exosome recognition and internalisation by recipient cells [29]. As such, exosomes may function as lipid carriers, allowing the transport of the bioactive lipids they carry to a recipient cell (see Section 4). This process of exosome trafficking, particularly in the context of tumour microenvironment, may lead to an enrichment of certain tumour progressive/immunosuppressive lipids, such as prostaglandins [30]. Conversely, it may also lead to a replacement of harmful exosome lipid contents with beneficial ones, such as docosahexaenoic acid, an omega-3 polyunsaturated fatty acid with many health and anticancer benefits, that could be supplied by exosomes throughout the tumour microenvironment affecting cell-to-cell communication, reducing tumour cell growth, and increasing sensitivity to therapeutic interventions [31].

### 3.3. Nucleic Acid Content of Exosomes

One of the most distinct features of exosomes is the fact that they carry significant amounts of nucleic acids, including microRNAs (miRNAs) and mRNA, as well as mitochondrial DNA (mtDNA), piwi-RNAs (piRNAs), long noncoding RNAs (lncRNAs), ribosomal RNAs (rRNAs), small-nuclear RNAs (snRNAs), small-nucleolar RNAs (snoRNAs), and transfer RNAs (tRNAs) [32]. Although the exosomal mRNA appeared to be mostly degraded to less than 200 fragments, it was possible the* in vitro* translation of full-length proteins [33, 34], suggesting that after being internalised by target cells, mRNA can be translated into proteins. The presence of miRNAs in exosomes has been subject of several studies [35–39]. miRNA content of exosomes has particular relevance in cancer pathologies, whose type and quantity of miRNAs vary with the cell of origin, and seems to reflect the miRNA content of the parental cells, although it was also found some discrepancies in exosomes derived from cancer cells, suggesting selective packaging of miRNAs [40]. mRNAs and miRNAs can be transferred to a recipient cell located in the tumour microenvironment or at distant sites via fusion of the exosome with the target cell membrane [7]. After internalisation by target cells these miRNAs may function as either tumour suppressors or oncogenes.

### 3.4. Others

Despite the relevance of glycosylation of the membrane surface in the communication of cells with their extracellular environment, very few studies have been made to characterize the carbohydrate content of exosomes. The analysis of glycosylation patterns of exosomes derived from T cells, melanoma, and colon cancer cells revealed that the glycosylation signature seems to be conserved between exosomes and parental cell membranes [41]. In another study, it was observed an enrichment of the sialoglycoprotein galectin-3-binding protein in ovarian tumour derived exosomes [42].

## 4. Exosomes Function

The major role of exosomes seems to be the transport of bioactive molecules between cells, with consequences in targeted cell phenotypes, such as mRNA and miRNA related to the transfer of genetic, and sometimes epigenetic, information between cells [13]. Additionally, and as described above, another exosome function includes lipid trafficking [30]. The presence of exosomes in healthy body fluids suggests a role of these vesicles in the normal physiology of the body, including communication in the immune system, tissue repair, and communication within the nervous system [43]. Exosomes have also been associated with infection [44] and several pathological conditions, such as in the progression of neurodegenerative disease, cardiovascular diseases and cancer [24], or, on the other side, in the protection against atherosclerosis [45, 46].

The importance of exosomes in tumorigenesis is emphasised by the general increased content of these vesicles in biological fluids of cancer patients relatively to healthy controls, being observed an increased content of exosomes as the tumour progresses [24]. Interestingly, CCEs cause both antitumorigenic and protumorigenic effects. Studies have shown that bladder cancer cell lines shed exosomes containing proteins important for tumour progression, and these exosomes inhibit tumour cell apoptosis through Akt and ERK pathways [47]. On the other side, CCEs can transport tumour antigens to dendritic cells and induce immune responses [24]. These differences between biological functions observed for exosomes most likely arise from differences in the cargo present either on the surface of the vesicle or internally.

Furthermore, CCEs have been implicated in tumour growth, survival, and spread, as well as in angiogenesis, escape from immune surveillance, stimulating tumour cell migration, conferring invasion ability to normal cells, and the preparation of distal tissues for their metastatic colonisation [13]. The following sections will focus on these subjects.

## 5. Exosomes Targeting and Uptake by Recipient Cells

The mechanisms underlying interaction and fusion of exosomes with target cells remain undefined. It is believed that the uptake of exosomes by target cells may occur through three main mechanisms: (i) simple fusion of the exosome with the cellular membrane, directly releasing the content of vesicles into the cytoplasm; (ii) exosome uptake by endocytosis; (iii) uptake dependent on the presence of distinct receptor proteins that enable binding of exosomes to target cells [32]. For the latter, it is generally accepted that the cell of origin and secretion conditions of exosomes seem to determine their cell surface content, and consequently the cell-type-specific adhesion molecules, targeting exosomes to specific cells [48]. Nevertheless, exosomes contain many different cell surface molecules and one single exosome is able to engage many different cell receptors [11, 48]. It was postulated that exosome recognition by cells involve lipid receptors, such as receptors of the TIM family that recognises PS and possibly a G protein coupled receptor family protein, G2A, that recognises LPC located at the surface of the vesicles [11]. Moreover, it is likely that exosomes attachment and internalisation are partially mediated by interactions with heparin sulphate proteoglycans (HSPG) located at the membrane of the receptor cell, possibly by a similar mechanism as for lipoprotein or virus internalisation [49].

## 6. Role of Exosomes in Tumour Microenvironment Development

As mentioned earlier, cancer development is a multistep process in which somatic cells experience events (such as environmental insults or chronic inflammation), accumulating genetic modifications that will ultimately result in uncontrolled growth of the cell [1]. During tumour development, the extracellular matrix suffers modifications that will support malignant progression. The evolution of the tumour microenvironment is driven (i) by the genetic instability of malignant cells, that are constantly releasing exosomes carrying oncogenes and other bioactive molecules involved in tumour progression; (ii) environmental selection forces, which include endogenous tumour-growth induced stress stimuli, such as hypoxia, acidosis, starvation, or oxidative stress; and (iii) inflammatory and immune responses [1].

**Figure 1:**
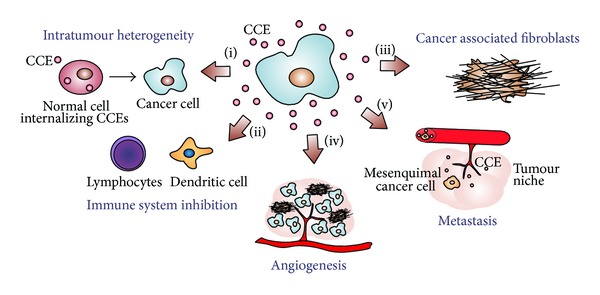
Tumour microenvironment processes mediated by cancer cell derived exosomes (CCEs): (i) intratumour heterogeneity resulting from phenotype modification of normal cells after internalization of CCEs; (ii) inhibition of the immune response against tumour cells by inhibiting the proliferative response of lymphocytes; (iii) activation of the differentiation of fibroblasts into cancer associated fibroblasts; (iv) stimulation of the angiogenic process; and (v) epithelial to mesenchymal transition (EMT) and preparation of a premetastatic niche at the distant location.

An epithelial tumour mass is composed by stromal elements that frequently include an altered extracellular matrix enriched with cytokines and growth factors, fibroblasts, a scaffold composed of immune and inflammatory cells, endothelial cells, pericytes, mesenchymal cells, and in the case of more advanced tumours, blood and lymph vessels and nerves [1]. Every stromal cell type seems to have the ability to support hyperproliferation of cancer cells in a specific context and seems to be different for each type of cancer pathologies [50, 51]. However, the molecular mechanisms related to the recruitment and maturation of stromal cells are not completely defined. Exosomes have important roles in the intercellular communication between cancer and stromal cells that will result in the maturation of the tumour microenvironment and tumour growth and proliferation (Figure 1).

### 6.1. Role of Exosomes in Intratumour Heterogeneity

A common trait between all tumour pathologies is the phenotypic heterogeneity of the population of cancer cells within tumours. This is mainly due to genetic instability of cancer cells leading to genetic alterations, differential environmental stimuli, and stochastic processes that occur within the tumour microenvironment [52]. It is likely that intratumour heterogeneity may also result from the internalisation of CCES by neighbour healthy epithelial cells. In fact, it was observed an alteration of the phenotype of normal cells after internalisation with exosomes derived from colorectal, lung, and prostate cancer cells [48, 53].

### 6.2. Role of Exosomes in Immunological Responses in Tumour Microenvironment

Generally, early tumour microenvironment resembles the environment of wounds that never heal [54]. In the context of immune response, the tumour microenvironment contains (i) innate immune cells, such as macrophages, neutrophils, mast cells, myeloid-derived suppressor cells, dendritic cells, and natural killer cells and (ii) adaptive immune cells, such as T and B lymphocytes. However the immune cells more represented in tumour microenvironment are tumour associated macrophages and T lymphocytes [55]. Exosomes play an important role in inter- and intracommunication between the immune cells and cancer cells.

As already referred, the communication between cells of the immunity system is mediated by direct contact or cytokine and chemokine production, which also involves exosomes [6]. It is the balance of the activation level and abundance of the immune mediators and modulators in the tumour microenvironment that dictates if the tumour-inflammatory response or antitumour immunity occurs [4]. Exosomes have important roles in this equilibrium. It was previously observed that, through a still not defined mechanism, exosomes derived from several tumours, including pleural malignant mesothelioma and prostate cancer, inhibited the proliferative response of lymphocytes or natural killer cells [43, 56]. Additionally, miRNA transported by CCEs may act like ligands by binding to Toll-like receptors and trigger the inflammatory response. In fact, it was observed that oncogene miR-21 and miR-29a secreted from exosomes derived from lung cancer cells were able to bind to murine and human TLR [57]. On the other way, exosomes may act in the specialised activation of T lymphocytes against cancer cells by the presentation of membrane proteins, such as HER2/Neu, enriched in tumour cells.

### 6.3. Role of Exosomes in Production of Cancer Associated Fibroblasts

Cancer associated fibroblasts (CAF) are the most prominent cell type in the tumour microenvironment of many cancers types, including colon, pancreas, and breast, and play critical roles in tumour-stromal interactions. The mechanisms inherent to CAF formation seem to involve the formation of myofibroblasts by differentiation of resident fibroblasts, epithelial and endothelial cells (via epithelial to mesenchymal transition (EMT)), pericytes, bone-marrow-derived circulating fibrocytes and mesenchymal stem cells [58]. This differentiation is mainly promoted by platelet derived- and ECCs containing-tumour growth factor-beta 1 (TGF-*β*1) and fibroblast growth factor-2 (FGF-2) [58, 59] and consists in a phenotypic change of the cell mediated by arrangements of the cytoskeleton, pericellular coats, extracellular matrix turnover, and growth factor production. Interestingly, Webber and collaborators observed that differentiation of fibroblasts into myofibroblasts only require cancer cells exosomal TGF-*β*, stressing the relevance of exosomes as effectors in the alteration of cancer stroma [59]. In addition, two independent studies revealed that exosomes derived from ovarian cancer cells are able to convert adipose tissue-derived mesenchymal stem cells into myofibroblasts-like cells [60, 61].

The altered phenotype of CAFs will result in the increased production of alpha smooth muscle actin with consequences in the increased stiffness of the extracellular matrix [58]. CAFs contribute to the architectural and molecular remodelling of the tumour microenvironment supporting tumour growth, vascularisation, and metastasis [58, 62, 63]. Indeed, as a consequence of the increased stiffness in the tumour microenvironment, epithelial cells may acquire a mesenchymal phenotype by losing the cell-cell junctions and cell polarity [58]. Furthermore, it is known that CAF release bioactive molecules, such as HGF, IL6, PDGF, prostaglandins, proteases and miRNAs to the extracellular matrix, suggesting a role of exosomes in their transport [62]. Additionally, Luga and Wrana observed that exosomes derived from CAFs promote activity, motility, and metastasis of breast cancer cells by activating autocrine Wnt-PCP signalling [63].

Another example of the role of CCEs in tumour progression is the release of exosomes containing the extracellular matrix MMPs inducer (EMMPRIN) by lung carcinoma cells [64]. The release of bioactive EMMPRIN stimulates the expression of the matrix MMPs in fibroblasts, with consequences in tumour metastization. In addition, exosomes released by prostate cancer cells under hypoxic conditions were loaded with a significant higher number of TGF-*β*, IL6, TNF-1a, and MMPs, that have been implicated in the induction of a stem cell phenotype in the microenvironment of tumour cells and promotion of metastasis [65].

### 6.4. Role of Exosomes in Angiogenesis

The vascular formation inherent to the cancer progression may be triggered by hypoxic and nutrient depletion conditions in the tumour microenvironment, as well as by inflammatory responses, usually observed in epithelial cell carcinomas [54]. The angiogenic process consists in a neovascular formation from preexisting blood vessels and results from numerous interactions between regulators, mediators, and stimulatory molecules. Endothelial cells and pericytes located at the tumour microenvironment are imperative in this process [66]. Vascular endothelial growth factors (VEGF), FGF, TGF-*β*, PDGF, and IL-8 are some of the angiogenic factors that act on the regulation of quiescence, migration, and proliferation of endothelial cells, required for the stimulation of angiogenesis [66].

Recent studies have shown that exosomes released under hypoxic conditions contribute to the stimulation of angiogenesis [66]. Melanoma cells derived exosomes containing miRNA-9 were internalised by endothelial cells promoting metastasis and angiogenesis by activation of the JAK-STAT pathway [32]. In another study, exosomes derived from metastatic breast cancer cells contained multiple angiogenic miRNAs, including miRNA-210 whose expression is inversely correlated with overall survival in breast cancer [35]. Moreover, it was observed that extracellular vesicles derived from hypoxic brain tumour glioblastoma multiform cells were enriched with angiogenic stimulatory molecules, such as IL-8 and PDGF [67].

### 6.5. Role of Exosomes in Metastasis

The increased malignancy of a tumour, with several implications on cancer patient survival, consists in the formation of tumour metastasis. The metastization process is complex and involves several steps including (i) EMT; (ii) breach of the basement membrane barrier; (iii) migration of the cell through the neighbouring tissue; (iv) entry, transport, and exit from blood and lymph vessels; (v) establishment of the cell in a secondary anatomical site; and (vi) growth of the secondary tumour, by the creation of a new tumour microenvironment favourable to cancer cell growth [3]. The role of exosomes in metastasis process relies mainly on the first, second, and, indirectly, the fifth steps.

During EMT, cancer cells loose the epithelial characteristics towards a more mesenchymal phenotype and acquire motility capabilities. The mechanisms inherent to this event are complex, involving cytoskeletal alterations and downregulation of expression of E-cadherin [65]. Interestingly, it was observed that proteins in exosomes derived from hypoxic prostate cancer cells are involved in the pathways of epithelial adherens junctions and cytoskeleton remodelling, suggesting that the increased invasiveness observed in prostate cancer cells is mediated by exosomes [65]. In another study, Jeppesen and collaborators [68] studied the protein content of exosomes derived from a human bladder carcinoma cell line without metastatic capacity relatively to two isogenic derivate metastatic cell lines formed in the lung and liver of mice. They reported an increased abundance in exosomes derived from metastatic cells, of vimentin, hepatoma-derived growth factor (HDGF), casein kinase II, and annexin A2, which are associated with the EMT process, as well as other proteins involved in cellular movement and cell-cell signalling.

Besides involvement in EMT, exosomes seem also to be involved in the formation and preparation of the premetastatic niche at the new anatomical location. Interestingly, it was reported that exosomes of melanoma cells are preferentially taken up by sentinel lymph nodes and prepare the premetastatic niche by deposition in the extracellular matrix and vascular proliferation in the lymph nodes. Subsequently, free melanoma cells were recruited to the lymph nodes that have taken up the cancer derived exosomes [69]. In another study, Peinado and collaborators suggested that melanoma derived exosomes induce vascular leakiness at premetastatic sites and increase the metastatic behaviour of bone marrow cells through the oncoprotein receptor kinase MET [70].

## 7. Clinical Relevance of Exosomes in Cancer

### 7.1. Use of Exosomes as Biomarkers

As already mentioned throughout this review, the content of an exosome depends on the cell of origin. Particularly, exosomes derived from cancer cells are enriched with proteins, mRNA, and miRNA that are more abundant in cancer cells than in normal cells [8]. Hence, exosomes may be used as biomarkers for cancer diagnosis. Several studies including proteomics and transcriptomics have been used to understand the content of exosomes derived from specific tumours, with the purpose of their use as biomarkers [36, 71, 72]. As an example Taylor and Gercel-Taylor observed that the levels of 8 microRNAs (miR-21, miR-141, miR-200a, miR-200b, miR-200c, miR-203, miR-205, and miR-214) are of diagnostic value in ovarian cancer [71]. They found that these microRNAs are present in circulating exosomes in the sera of patients and the levels of the microRNAs found in these vesicles were similar to those found in the cancer cells, suggesting an effective way to diagnose ovarian cancer even in asymptomatic patients [71].

One of the major advantages of the use of exosomes as biomarkers is the possibility of a rapid pathology prognosis through a minimal invasive procedure. In fact, one of the higher challenges in the battle against tumours is an early and accurate diagnosis [73]. Currently, the tissue biopsy, which is an invasive procedure, with potential damaging side effects, is generally required for a correct diagnosis. The presence in body fluids of exosomes containing biomarkers of subtypes of cancer cells may allow the use of minimal invasive “liquid biopsies” (such as blood collection) for prognosis and diagnosis of cancer. In contrast to the circulating tumour cells (CTCs), which were also suggested as important biomarkers for real-time diagnosis of cancer progression, the modification of the content of exosomes accompanies the development of the tumorigenicity of the cells, allowing the prognosis of cancer since the beginning of the pathology. However, the quantity and heterogeneity of exosomes in body fluids may be a drawback in the use of these vesicles as biomarkers, as it can lead to false negatives or positives in prognosis and diagnosis [73].

### 7.2. Use of Exosomes for Cancer Therapeutics

**Figure 2:**
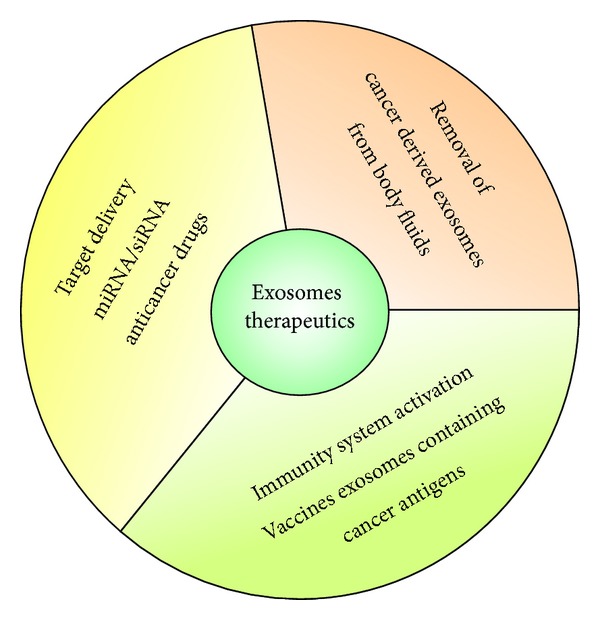
Major groups of exosomes based therapies, which include the removal of cancer derived exosomes, containing bioactive molecules, from the blood (or other body fluids) of cancer patients through a haemodialysis-like process; activation of the immune system against cancer cells by the use of vaccines of exosomes containing proteins with higher expression in tumour cell membranes; and use of exosomes containing microRNA (miRNA), small interference RNA (siRNA), and/or anticancer drugs for targeting delivery to cancer cells.

From a therapeutically point of view, the natural role of exosomes as carriers of metabolites from donor cells to recipient cells and in inducing a biological response has been investigated by several researchers in a dual manner. Exosomes may be used both as mediators of tumour resistance and as vessels for targeted drug delivery.  Figure 2 highlights the therapies that were proposed for treatment of tumours based on exosomes characteristics.

Several studies suggested a role of exosomes in drug resistance, by extruding hydrophilic drugs from cancer cells [74, 75], in resistance to radiation [76] and to immunotherapy [77, 78]. To surpass this negative effect of exosomes in cancer treatment, the removal of exosomes from the blood circulation of patients in a haemodialysis-like procedure was proposed [79].

On the other side, since the first suggestions of the role of exosomes in the immune system performed by Raposo et al. [80] and Zitvogel et al. [81] that exosome based cell-free vaccines could represent an alternative to dendritic cell therapy for suppressing tumour growth. With this in mind, three phase I [82–84] clinical trials were performed using dendritic and ascites derived exosomes. However, it was observed that only a small percentage of patients presented transient stabilisation of the disease. A phase II clinical trial was also described in 2009 that combined the administration of dendritic cell derived exosomes carrying NKg2D ligands and Il-15R*α*, in association with Treg cell-inhibiting treatments in patients with non-small-cell lung cancer that has been stabilised by chemotherapy [85].

One other use of exosomes for cancer therapy is the possibility of using these vesicles for targeted delivery. This process may be accomplished by the targeting of cancer cells mediated by specific antibodies or ligands of highly expressed membrane receptors, further internalisation of exosomes and induced apoptosis of the cells, which may be mediated by miRNA, siRNA and anticancer drugs. Several studies have described the successful delivery and tumour inhibition using this process [86–88]. As an example, Alvarez-Erviti et al. [87] developed a way to introduce siRNA into exosomes by electroporation and to specifically target these siRNA loaded exosomes to neurons. In another study published in 2013, Ohno et al. [88] successfully delivered let-7a miRNA to xenografted breast cancer cells in mice.

## 8. Conclusions and Future Directions

Current knowledge of CCEs suggests that they can play an important role in the development and progression of cancer through modulation of intercellular communication within the tumour microenvironment by the transfer of protein, lipid, and RNA cargo. The complete understanding of their role in intercellular communication and tumorigenesis will be achieved by further exploration and comparison of their secretion in normal cells and during cancer development and progression, together with assessment of their specific content under these conditions. Exploration of CCEs contents may allow the development of novel diagnostic and therapeutic approaches, with minimally invasive procedures.

The use of exosomes for targeted delivery may also prove to be the answer in iRNA based therapeutics, whose main drawback has been the development of an effective delivery system. Generation of synthetic exosomes—“exosome mimetics”—for drug delivery may also allow selective targeting of cancer cells (for a review see [86]). Although in its infancy, the future possibilities of these natural nanovesicules are tremendous.

## Abbreviations

CAF: Cancer associated fibroblasts
CCEs: Cancer cells derived exosomes
EMT: Epithelial to mesenchymal transition.
